# Supplementary material for: Impedimetric Determination of Kanamycin in Milk with Aptasensor Based on Carbon Black-Oligolactide Composite
Source: Sensors (Basel). 2020 Aug 21;20(17):4738. doi: 10.3390/s20174738 (PMC7506709; doi:10.3390/s20174738)
Supplement: Supplementary file 1 [file sensors-20-04738-s001.pdf]

sensors

MDPI

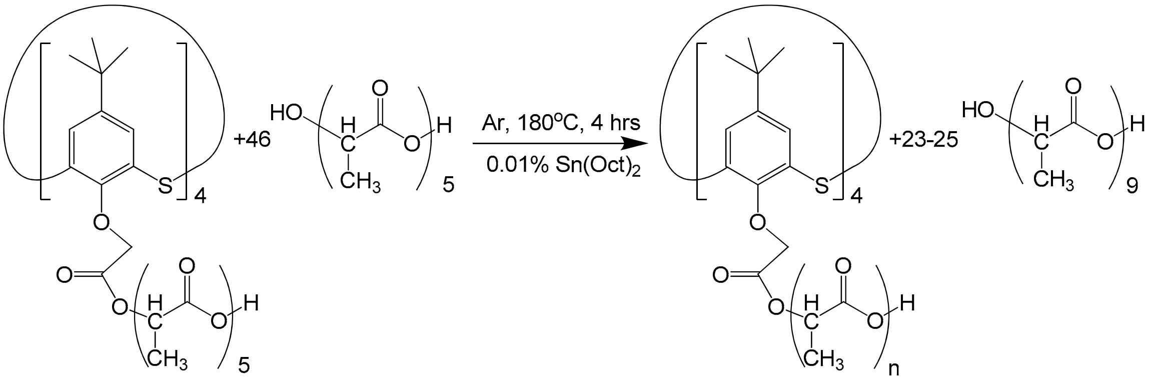


**Scheme S1.** General scheme of the synthesis of thiacalix[4]arene bearing oligolactide fragments (OLA-*cone*).

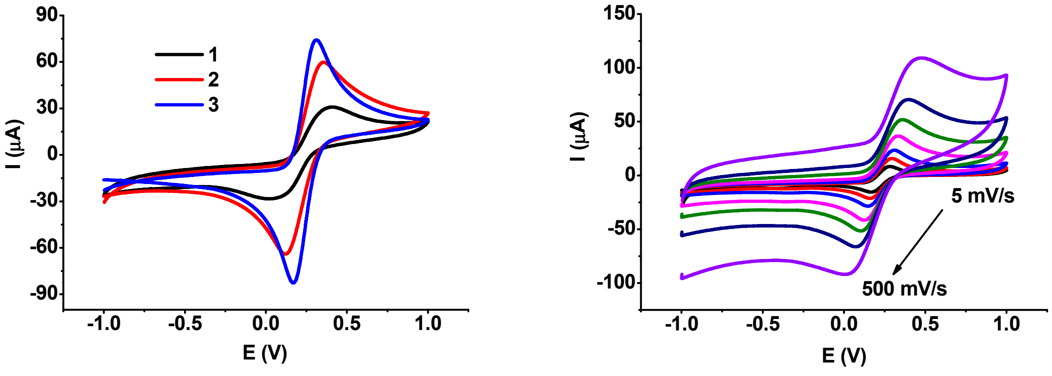


(**a**) (**b**)

**Figure S1.** Cyclic voltammograms recorded with the glassy carbon electrode (GCE) covered with the carbon black (CB), OLA-*cone*, and aptamers. Measurements in phosphate buffer (PB), pH = 7.0, in the presence of 0.01 M $K_3[Fe(CN)_6]$ and 0.01 M $K_4[Fe(CN)_6]$ at the scan rate of 100 mV/s. (**a**) 1: CB in chitosan matrix; 2: CB in chitosan matrix mixed with the OLA-*cone*/aptamer 1:1 mixture; 3: the same aptasensor after 20 min incubation in 1.0 nM KANA; (**b**) CB in chitosan matrix mixed with the OLA-*cone*/aptamer 1:2 mixture, depending on the scan rate.

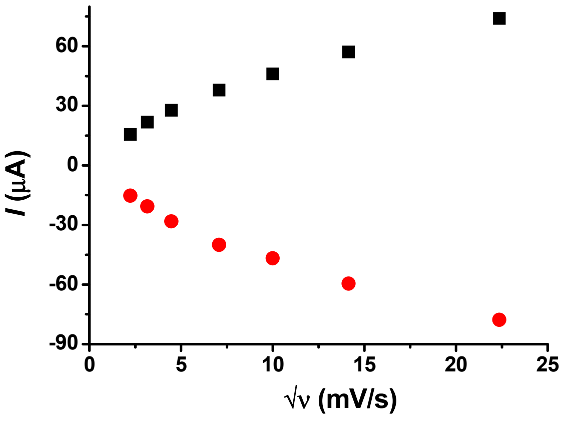


**Figure S2.** The dependence of the cathodic (black squares) and anodic (red circles) peak currents of cyclic voltammograms recorded with the GCE covered with the CB/chitosan and OLA-*cone*/aptamer (1:2) suspension on the square root from the scan rate, mV/s. Measurements in PB, pH = 7.0, in the presence of 0.01 M $K_3[Fe(CN)_6]$ and 0.01 M $K_4[Fe(CN)_6]$.

**Table S1.** The dependence of the relative increase of the working surface area of the electrode (n) calculated from the Randles–Sevcik equation on the pH and concentration of the PB. Deposition of 2 μL of 1 mg/mL suspension of the CB in 0.275% chitosan.

| PB Concentration (mM) | pH | Real Electrode Surface ($mm^2$) | n |
|---|---|---|---|
| 25 | 6.0 | 2.50 | 1.3 |
| 25 | 7.0 | 2.54 | 1.3 |
| 25 | 7.4 | 2.53 | 1.3 |
| 25 | 8.0 | 2.55 | 1.3 |
| 10 | 7.0 | 2.53 | 1.3 |
| 35 | 7.0 | 2.54 | 1.3 |
| 50 | 7.0 | 2.56 | 1.3 |

**Table S2.** The dependence of the electrochemical impedance spectroscopy (EIS) parameters ($(R_{et})_1$, $CPE_1$, and $n_1$) on the period of incubation in 1.0 nM Kanamycin A (KANA) solution. Aptasensor with the CB/chitosan and the OLA-*cone*/aptamer 1:2 mixture. Electrode–layer interface, average ± standard deviation for three measurements with individual aptasensors. $(R_{et})_1$ is the charge transfer resistance, $CPE_1$ is the constant phase element and $n_1$ is exponent in Equation (6) of the main text.

| Incubation Period (min) | $(R_{et})_1$ (kΩ) | $CPE_1$ (μF) | $n_1$ |
|---|---|---|---|
| 10 | 0.52 ± 0.02 | 1.50 ± 0.10 | 0.95 |
| 20 | 0.43 ± 0.03 | 1.41 ± 0.22 | 0.93 |
| 30 | 0.42 ± 0.01 | 1.32 ± 0.11 | 0.02 |
| 40 | 0.41 ± 0.02 | 1.44 ± 0.09 | 0.93 |

© 2020 by the authors. Licensee MDPI, Basel, Switzerland. This article is an open access article distributed under the terms and conditions of the Creative Commons Attribution (CC BY) license (http://creativecommons.org/licenses/by/4.0/).
